# Association of Neuropeptide-Y (*NPY*) and Interleukin-1beta (*IL1B*), Genotype-Phenotype Correlation and Plasma Lipids with Type-II Diabetes

**DOI:** 10.1371/journal.pone.0164437

**Published:** 2016-10-17

**Authors:** Roma Patel, Mitesh Dwivedi, Mohmmad Shoab Mansuri, Ansarullah, Naresh C. Laddha, Ami Thakker, A. V. Ramachandran, Rasheedunnisa Begum

**Affiliations:** 1 Department of Biochemistry, Faculty of Science, The Maharaja Sayajirao University of Baroda, Vadodara, Gujarat, India; 2 Department of Zoology, Faculty of Science, The Maharaja Sayajirao University of Baroda, Vadodara, Gujarat, India; Mathematical Institute, HUNGARY

## Abstract

**Background:**

Neuropeptide Y (NPY) is known to play a role in the regulation of satiety, energy balance, body weight, and insulin release. Interleukin-1beta (IL1B) has been associated with loss of beta-cell mass in type-II diabetes (TIID).

**Objectives:**

The present study attempts to investigate the association of *NPY* exon2 +1128 T/C (Leu7Pro; rs16139), *NPY* promoter -399 T/C (rs16147) and *IL1B* -511 C/T (rs16944) polymorphisms with TIID and their correlation with plasma lipid levels, BMI, and *IL1B* transcript levels.

**Methods:**

PCR-RFLP was used for genotyping these polymorphisms in a case-control study involving 558 TIID patients and 1085 healthy age-matched controls from Gujarat. Linkage disequilibrium and haplotype analysis of the *NPY* polymorphic sites were performed to assess their association with TIID. *IL1B* transcript levels in PBMCs were also assessed in 108 controls and 101 patients using real-time PCR.

**Results:**

Our results show significant association of both structural and promoter polymorphisms of *NPY* (*p*<0.0001 and *p*<0.0001 respectively) in patients with TIID. However, the *IL1B* C/T polymorphism did not show any association (*p* = 0.3797) with TIID patients. Haplotype analysis revealed more frequent association of CC and CT haplotypes (*p* = 3.34 x 10^−5^, *p* = 6.04 x 10^−9^) in diabetics compared to controls and increased the risk of diabetes by 3.02 and 2.088 respectively. Transcript levels of *IL1B* were significantly higher (*p*<0.0001) in patients as compared to controls. Genotype-phenotype correlation of *IL1B* polymorphism did not show any association with its higher transcript levels. In addition, *NPY* +1128 T/C polymorphism was found to be associated with increased plasma LDL levels (*p* = 0.01).

**Conclusion:**

The present study provides an evidence for a strong correlation between structural and promoter polymorphisms of *NPY* gene and upregulation of *IL1B* transcript levels with susceptibility to TIID and altering the lipid metabolism in Gujarat population.

## Introduction

Type-II diabetes (TIID) is a multifactorial disorder characterized by chronic hyperglycemia, insulin resistance and impaired insulin secretion and/or action. Sedentary lifestyle and high carb diet which leads to obesity are the contributing factors for lifestyle disorder “TIID” [1]. According to the estimates of International Diabetes Federation (IDF) and World Diabetes Foundation (WDF), India has the second largest diabetic population in the world i.e., ~62 million. In terms of regional prevalence, Gujarat has the largest number of diabetic population according to national health profile (2015) by Central Bureau of Health Intelligence (CBHI). Despite the fact that non-genetic or environmental factors contribute to ethnic variability, substantially varied prevalence among ethnic groups attest to the contribution of genetic factors in predisposition to diabetes [2]. We previously reported the association of angiotensin converting enzyme (*ACE)* I/D polymorphism with TIID in Gujarat population, suggesting a possible genetic link with the disease [3].

Neuropeptide Y (NPY) and interleukin 1 beta (IL1B) play important roles in insulin resistance and impairment. The human *NPY* gene contains two well-known polymorphisms: promoter region variation -399 (rs16147) and a non-synonymous variation +1128 SNP (rs16139). Previously, Sommer et al. [4] showed promoter polymorphism to result in elevated expression of NPY. Earlier studies have also shown +1128 T/C polymorphism of preproNPY to be associated with increased risk for vascular complications in TIID [5]. NPY is a well characterized 36-amino acid neuromodulator secreted by neurons in the central and peripheral nervous systems. The *NPY* gene is located on chromosome 7 and is about 8 kb in length with four exons separated by three introns [6–7]. Karvonen et al. [8] first reported +1128 T>C SNP that causes an amino acid change from Leucine to Proline (Leu7Pro) in the signal peptide of NPY to be associated with high serum cholesterol and LDL cholesterol levels. This polymorphism was further found to be associated with diabetic retinopathy in TIID [9]. Another SNP (rs16147) -399 T/C in NPY gene alters its *in vitro* expression and possibly is responsible for *in vivo* change in mRNA expression levels [4, 10]. It has been shown that an anxiolytic peptide—NPY is responsible for inter-individual variation pliable to stress and thus poses a risk for a number of diseases [10].

The *IL1B* gene located on chromosome 2 and encoding a protein of 269 amino acids is a chief regulator of the body’s inflammatory response and is produced consequent to injury and antigenic challenge. IL1B is known to exert various biological effects. It has been implicated in a range of autoimmune diseases such as rheumatoid arthritis, inflammatory bowel diseases, and type-I diabetes, as well as in diseases linked to metabolic syndromes such as TIID, atherosclerosis, and chronic heart failure [11]. Previously, Rosmaninho-Salgado et al. [12] showed the involvement of IL1B in the induction of NPY release.

The present study was aimed to deduce whether i) the two well-characterized *NPY* polymorphisms [exon 2 +1128T/C (rs16139) and -339T/C (rs16147)] and *IL1B* promoter polymorphism -511C/T (rs16944) are associated with susceptibility to TIID in Gujarat population; ii) the genotype-phenotype correlation of above-mentioned SNPs is associated with TIID, and iii) the *NPY* and *IL1B* polymorphisms play a significant role in altering the lipid metabolism in patients.

## Materials and Methods

### Study Subjects

This study was approved by Institutional Ethical Committee for Human Research (IECHR), Faculty of Science, The Maharaja Sayajirao University of Baroda, Vadodara, Gujarat, India (FS/IECHR/2013/1). The importance of the study was explained to all participants and written consent was obtained from all patients and control subjects. The study group included 558 TIID patients (293 males and 265 females) and 1085 non-diabetic subjects (553 males and 532 females) as shown in S1 Table. The TIID subjects recruited for the study displayed fasting blood glucose levels (FBS) >125 mg/dl. BMI (weight kg/height m^2^) was calculated by measuring height and weight.

### Blood collection, DNA extraction and Lipid Profiling

Three ml venous blood was collected from diabetic and ethnically matched non-diabetic individuals in K_3_EDTA coated tubes (Greiner Bio-One, North America Inc., North Carolina, USA). Plasma was separated and stored at -20°C for lipid profile estimation. FBS, total cholesterol (TC), triglycerides (TG), high-density lipoprotein (HDL) was analyzed using a commercial kit (Reckon Diagnostics P. Ltd, Vadodara, India). Low-density lipoprotein (LDL) was calculated using Friedewald’s (1972) formula. DNA was extracted from the whole blood using a QIAamp DNA Blood Mini Kit (Qiagen, Germany). The DNA content and purity were determined spectrophotometrically by 260/280 ratio. The integrity of DNA was checked electrophoretically using 0.8% agarose gel. The DNA was stored at -20°C until further analysis.

### Genotyping of *NPY* and *IL1B* SNPs by PCR-RFLP

*NPY* and *IL1B* SNP genotyping were done by using polymerase chain reaction-restriction fragment length polymorphism (PCR-RFLP) method. The primers used for genotyping of these polymorphisms are mentioned in S2 Table.

Twenty μl of the reaction mixture included 3 μl (50 ng) of genomic DNA, 11 μl H_2_O, 2.0 μl of 10X PCR buffer, 2.0 μl of 2.5 mM dNTPs (SIGMA Chemical Co, St. Louis, Missouri, USA), 1.0 μl each of 10 μM forward and reverse primers (MWG Biotech, India) and 0.3 μl of 3U/μl Taq Polymerase (Bangalore Genei, India). Amplification was performed using an Eppendorf Mastercycler gradient (USA Scientific, Inc., Florida, USA) as per the protocol of initial denaturation at 95°C for 5 minutes followed by 39 cycles each at 95°C for 30 seconds, 61°C for 30 seconds (primer specific; S2 Table), and 72°C for 30 seconds, followed by final extension at 72°C for 10 minutes. The amplified products were analyzed by electrophoresis on a 2.0% agarose gel and stained with ethidium bromide. The gel was visualized under a UV transilluminator with a 100 bp DNA ladder (MBI Fermentas, St. Leon-Rot, Germany) and photographed.

PCR-RFLP method was used for genotyping of all the three SNPs. The PCR product for genotyping of rs16139: T>C was subjected to restriction digestion using *BseN*I (MBI Fermentas, St. Leon-Rot, Germany) enzyme for 16 h at 65°C. The digested products (379 bp and 23 bp) were resolved on 20% polyacrylamide gel containing 0.5 μg/ml ethidium bromide. The PCR amplicon of the promoter region (rs16147: T>C) was subjected to restriction digestion using *Alu*I enzyme for 16 h at 37°C. The digested products (282 bp and 196 bp) were resolved on a 2.0% agarose gel containing 0.5 μg/ml ethidium bromide. A 100-bp DNA ladder (MBI Fermentas, St. Leon-Rot, Germany) was used as a marker for each gel. Similarly, the PCR products for genotyping of rs16944: C>T were subjected to restriction digestion using *Bsu36I* (MBI Fermentas, St. Leon-Rot, Germany) enzyme for 16 h at 37°C. The digested products (192 bp and 113 bp) were resolved on a 2.0% agarose gel containing 0.5 μg/ml ethidium bromide. All the gels were visualized under UV transilluminator using a gel visualizing system (Alpha Imager HP, Alpha Innotech Corporation, San Leandro, CA). More than 10% of the samples were randomly selected for genotype confirmation and the results showed 100% concordance (analysis of the chosen samples by two researchers independently) and further confirmed by DNA sequencing.

### Determination of *IL1B* mRNA expression

#### RNA isolation and cDNA synthesis

Total RNA from whole blood was isolated using Ribopure blood Kit (Ambion Inc. Texas, USA) by following the manufacturer’s protocol. RNA integrity was verified by gel electrophoresis using 1.5% agarose, RNA purity and yield was determined spectrophotometrically at 260/280 nm. RNA was treated with DNase I (Ambion Inc. Texas, USA) before cDNA synthesis to avoid DNA contamination. One microgram of total RNA was used to prepare cDNA using the RevertAid First Strand cDNA Synthesis Kit (Fermentas, Vilnius, Lithuania) according to the manufacturer’s instructions in the Eppendorf Mastercycler gradient (USA Scientific, Inc., Florida, USA).

#### Real-time PCR

*IL1B* and *GAPDH* (reference) transcripts were estimated by quantitative PCR using SYBR Green method and their respective forward and reverse primers (Eurofins, Bangalore, India) as shown in S2 Table. Real-time PCR (LightCycler480 Real- Time PCR, Roche) was performed in duplicate in 10 μl volume using LightCycler480 SYBR Green I Master mix (Roche Diagnostics GmbH, Mannheim, Germany) as per the instruction manual. The thermal cycling conditions included an initial activation step at 95°C for 10 min, followed by 45 cycles of denaturation, annealing, and amplification (95°C for 10 sec., 69°C for 10 sec (primer specific)., 72°C for 10 sec.). The fluorescence data collection was performed during the extension step. At the end of the amplification phase, a melt curve analysis was carried out to validate the specificity of the products formed. The PCR cycle at which PCR amplification begins its exponential phase was considered as the crossing point (Cp) or cycle threshold (Ct). The ΔCt or ΔCp value was obtained as a difference between the Ct of a target gene (*IL1B*) and Ct of reference gene (GAPDH). The difference among the two ΔCt values (ΔCt Controls and ΔCt patients) was considered as ΔΔCt to attain the value of fold expression (2^-ΔΔCt^).

### Statistical analysis

To test the genetic equilibrium of the populations, Hardy-Weinberg analysis was carried out by applying the equation (p^2^+2pq+q^2^) while for deviation from Hardy-Weinberg equilibrium (HWE) in controls and patients, the chi-square goodness-of-fit test was used.

The distribution of the genotypes and allele frequencies of *NPY* polymorphism for patients and control subjects was compared using the chi-square test with 3×2 and 2×2 contingency tables respectively using GraphPad Prism version 3.00 for Windows (GraphPad Software, San Diego California, USA). Odds ratio (OR) with respective confidence interval (95% CI) for disease risk was also calculated. *p*-values less than 0.05 were considered as statistically significant.

Haplotype analysis was carried out using http://analysis.bio-x.cn/myAnalysis.php [13]. The linkage disequilibrium (LD) coefficients D’ (D/Dmax) and r^2^ values for the pair of the most frequent alleles at each site were estimated using the Haploview program version 4.1 [14]. The statistical power of detection of the association with the disease at the 0.05 level of significance was determined by using the G* Power software [15].

Relative expression of *IL1B* in patient and control groups was analyzed by GraphPad Prism version 3.00 for Windows (GraphPad Software, San Diego California, USA). Cochran-Armitage trend test was performed using SAS 9.2 software for analyzing *IL1B* transcript levels with respect to the genotype for each group individually [16]. Further, ANOVA’s trend test was used to evaluate the mean ΔCp values for different genotypes in patients and controls using SPSS version 20 software. The relative gene expression of *IL1B*, FBS levels, BMI and lipid profile in patient and control groups were analyzed by nonparametric unpaired t-test.

## Results

### Analysis of association between *NPY* gene exon 2 +1128T/C polymorphism and susceptibility to type-II diabetes

**Fig 1:**
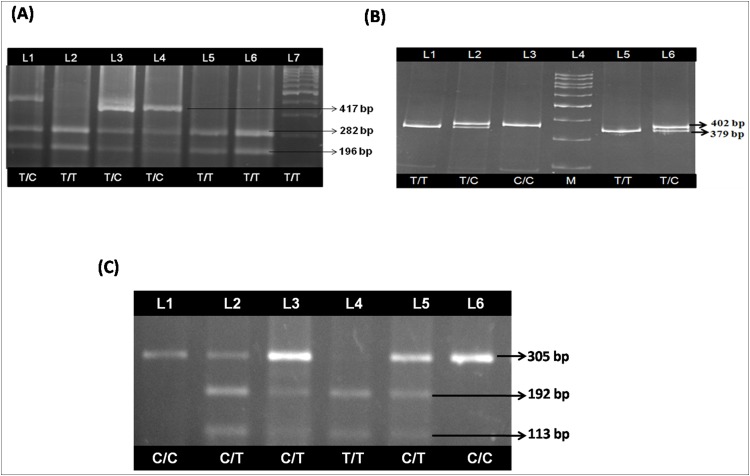
PCR-RFLP analysis of *NPY* exon 2 (+1128; T/C), *NPY* promoter (-399; T/C) and *IL1B* promoter (-511; C/T) polymorphisms. **(A)** PCR-RFLP analysis of *NPY* exon 2 (+1128; T/C) polymorphism on 2.0% agarose gel: lanes: 1 and 5 show homozygous (TT) genotypes; lane: 2 and 6 show heterozygous (TC) genotypes; lane: 3 shows homozygous (CC) genotype; lane: 4 shows 100 bp DNA ladder. **(B)** PCR-RFLP analysis of *NPY* promoter (-399; T/C) polymorphism on 3.5% polyacrylamide gel: lanes: 1, 3 and 4 show heterozygous (TC) genotypes; lanes: 2, 5 and 6 show homozygous (TT) genotypes; lane: 3 shows homozygous (TT) genotype; lane: 7 shows 100 bp DNA ladder. **(C)** PCR-RFLP analysis of *IL1B* promoter (-511; C/T) polymorphism on 2.0% agarose gel: lanes: 1 and 6 show homozygous (CC) genotypes; lanes: 2, 3 and 5 show heterozygous (CT) genotypes; lane: 4 shows homozygous (TT) genotype.

PCR-RFLP for +1128T/C polymorphism yielded a 402 bp undigested product corresponding to C allele and 379 bp and 23 bp digested products corresponding to T allele. The three genotypes identified by 20% polyacrylamide gel electrophoresis were: TT homozygous, TC heterozygous and CC homozygous for +1128T/C polymorphism of *NPY* gene (Fig 1A).

Exon 2 +1128T/C polymorphism of *NPY* gene was found to be associated with TIID patients (*p*<0.0001) when genotypes were compared with chi-square test-3x2 contingency table. Further, there was a significant difference in allele frequencies for this polymorphism between patients and controls when compared with 2x2 contingency table (*p*<0.0001). The control and patient groups showed deviation from the Hardy-Weinberg Equilibrium (HWE) (*p* = 0.0226 and *p*<0.0001 respectively). Moreover, there was a significant difference between genotype frequencies (TT vs TC and TT vs CC) in controls and patients (*p* = 0.0006, *p*<0.0001 respectively) as shown in Table 1.

**Table 1:** Association studies for *NPY* exon 2 (+1128; T/C), promoter (-399; T/C) and *IL1B* promoter (-511; C/T) polymorphisms in diabetic patients and controls from Gujarat population.

Gene/ SNP	Genotype or allele	Controls (Frequency)	Patients (Frequency)	*p* for Association	Odds ratio	(95% CI)
*NPY* Exon 2 +1128 T/C (rs16139)	**Genotype**	**(n = 1085)**	**(n = 558)**			
	TT	998	452	R	-	-
	TC	82	67	0.0006^a^	1.804	1.282 to 2.538
	CC	05	39	<0.0001^b^	17.22	6.742 to 43.99
	**Allele**					
	T	2078 (0.96)	971 (0.87)	<0.0001^c^	3.373	2.569 to 4.428
	C	92 (0.04)	145 (0.13)			
*NPY* -399 T/C (rs16147)	**Genotype**	**(n = 1000)**	**(n = 497)**			
	TT	314	196	R	-	-
	TC	471	159	<0.0001^a^	0.5408	0.4198 to 0.6967
	CC	215	142	<0.0001^b^	0.4403	0.3410 to 0.5686
	**Allele**					
	T	1099 (0.55)	551 (0.55)	0.8026^c^	0.9807	0.8415 to 1.143
	C	901 (0.45)	443 (0.45)			
*IL1B* -511 C/T (rs16944)	**Genotype**	**(n = 889)**	**(n = 556)**			
	CC	167	120	R	-	-
	CT	463	286	0.8596^a^	0.6516	0.6516 to 1.134
	TT	259	150	0.2089^b^	0.1598	0.1598 to 0.2731
	**Allele**					
	C	797 (0.45)	526 (0.47)	0.3797^c^	0.9051	0.7788 to 1.052
	T	981 (0.55)	586 (0.53)			

‘n’ represents number of Patients/ Controls, CI represents Confidence Interval

R refers to reference

^a^ and ^b^ represents Patients versus Controls (genotype) *p*-value for TT versus TC and TT versus CC of *NPY* +1128 T/C and *NPY* -399 T/C and respectively for CC versus CT and CC versus TT of *IL1B* -511 C/T.

^c^ represents Patients versus Controls (allele) *p*-value

Statistical significance was considered at *p*-value ≤ 0.05.

### Analysis of association between *NPY* gene promoter -339T/C polymorphism and susceptibility to type-II diabetes

The genotyping of -339T/C polymorphism of *NPY* revealed a 417 bp undigested product corresponding to C allele and 282 bp and 196 bp digested products corresponding to T allele by PCR-RFLP method. The three genotypes identified by 2.0% agarose gel electrophoresis were: TT homozygous, TC heterozygous and CC homozygous for -339T/C polymorphism of *NPY* gene (Fig 1B).

The promoter -339T/C polymorphism of *NPY* was found to be significantly associated with TIID patients (*p*<0.0001) when genotypes were compared using chi-square test-3x2 contingency table. However, there was no significant difference in allele frequencies of this polymorphism between patients and controls when compared with 2x2 contingency table (*p* = 0.8026). The control group was found to be in HWE for this polymorphism, however, the patient group deviated from the HWE (*p* = 0.1237 and *p*<0.0001 respectively). Further, there was a significant difference between genotype frequencies (TT vs TC and TT vs CC) in controls and patients (*p*<0.0001, *p*<0.0001 respectively) (Table 1).

### Linkage disequilibrium (LD) and haplotype analysis

The LD analysis of the two polymorphisms investigated in *NPY* revealed low LD association (+1128 T/C: -399 T/C; D’ = 0.325, r^2^ = 0.006). A haplotype assessment of the two polymorphic sites was performed and the estimated frequencies of the haplotypes differed significantly between TIID patients and controls (global *p*-value = 2.05 x 10–11). However, the CC haplotype was more frequently observed in diabetic patients and increased the risk of diabetes by 3-fold [*p* = 3.34 x 10^−5^; odds ratio (OR): 3.028; 95% confidence interval (CI): (1.750–5.240)] (Table 2). In addition, the CT haplotype was also frequently observed in diabetic patients and increased the risk of diabetes by 2.9-fold [*p* = 6.04 x 10–9; odds ratio (OR): 2.888; 95% confidence interval (CI): (1.991–4.189)] (Table 2).

**Table 2:** Distribution of haplotype frequencies for *NPY* exon2 (+1128; T/C) and promoter (-399; T/C) polymorphisms between diabetic patients and controls from Gujarat population.

Haplotype (+1128 T/C and -399 T/C)	Controls (Freq.%) (n = 1085)	Patients (Freq.%) (n = 558)	*p* for Association	*p*_(global)_	Odds ratio (95%CI)
CC	22.10 (1.2%)	31.83 (3.4%)	3.34 x 10^−5^	2.05 x 10^−11^	3.028 [1.750–5.240]
CT	50.90 (2.7%)	68.17 (7.3%)	6.04 x 10^−9^		2.888 [1.991–4.189]
TC	876.90 (45.8%)	388.17 (41.6%)	0.035826		0.844 [0.721–0.989]
TT	964.10 (50.4%)	443.83 (47.6%)	0.168597		0.896 [0.766–1.048]

n represents number of Patients/ Controls, CI represents Confidence Interval.

### Analysis of association between *IL1B* gene promoter -511 C/T polymorphism and susceptibility to type-II diabetes

The genotyping of -511C/T polymorphism of *IL1B* revealed a 305 bp undigested product corresponding to C allele and 192 bp and 113 bp digested products corresponding to T allele by PCR-RFLP method. The three genotypes identified by 2.0% agarose gel electrophoresis were: CC homozygous, CT heterozygous and TT homozygous for -511C/T polymorphism of *IL1B* gene (Fig 1C).

The allele and genotype frequencies for *IL1B* -511C/T polymorphism were calculated in diabetic and non-diabetic subjects. No difference in the genotype and allele frequencies was observed between diabetic and non-diabetic subjects (*p* = 0.3797, *p* = 0.1935 respectively). The control and diabetic group were found to be in HWE for this polymorphism, (*p* = 0.1148 and *p* = 0.4536 respectively). Also, there was no difference between genotype frequency TT vs TC while TT vs CC frequency differed significantly in controls and patients (*p* = 0.2844, *p*<0.0001 respectively) (Table 1).

### Relative gene expression of *IL1B* in TIID patients and controls

**Fig 2:**
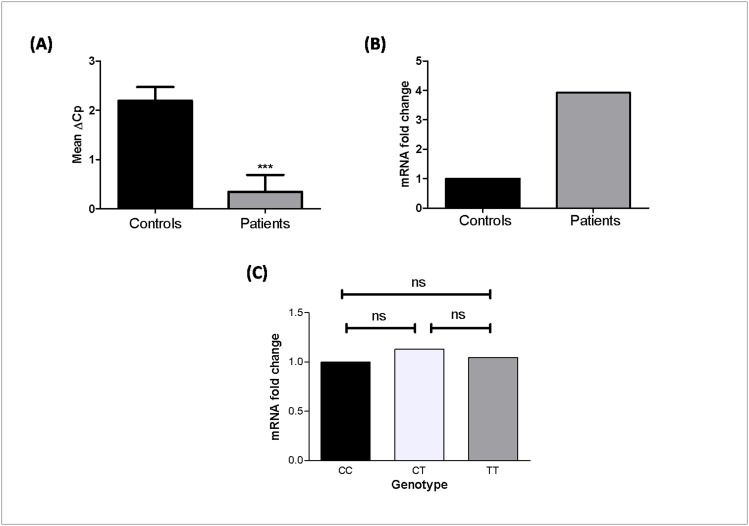
Relative gene expression of *IL1B* in controls and TIID patients. **(A)** Expression of *IL1B* transcripts in 108 controls, 101 TIID patients, as suggested by Mean ΔCp. TIID patients showed significantly increased mRNA levels of *IL1B* as compared to controls (Mean ΔCp ± SEM: 2.197 ± 0.2777 vs 0.2286 ± 0.3209; p<0.0001). **(B)** Expression fold change of *IL1B* transcripts in 108 controls and 101 TIID patients showed 3.92 fold change as determined by 2^-ΔΔCp^ method. **(C)** Expression fold change of *IL1B* transcripts with respect to genotypes of *IL1B* C/T (rs16944) promoter polymorphism in individuals having n = 28 CC, n = 95 CT, and n = 49 TT. There was no significant difference between CC vs CT genotype, CC vs TT genotype and CC vs CT vs TT genotype (p = 0.8043, p = 0.8403 and p = 0.9585 respectively) [ns = non-significant].

The *IL1B* transcript levels were monitored in 101 diabetic patients and 108 age-matched controls after normalization with *GAPDH* transcript levels. The *IL1B* transcript levels in diabetic patients were significantly higher than in controls (*p*<0.0001) as suggested by mean ΔCp values (Fig 2A). The 2^-ΔΔCp^ analysis showed about 4 fold higher expression of *IL1B* transcript in patients as compared to controls (Fig 2B).

### Correlation of *IL1B* transcript levels with -511 C/T promoter polymorphism

Expression of *IL1B* with respect to its promoter polymorphism (rs16944) revealed that there was no significant difference in the expression of *IL1B* between individuals with different genotypes (*p* = 0.9585) (Fig 2C). There was no difference in the expression of *IL1B* between individuals with CC and CT and, CC and TT genotype (*p* = 0.8043, *p* = 0.8403 respectively). Moreover, ANOVA’s trend test was used to see the change in mean ΔCp values across the different -511 C/T promoter genotypes. The analysis revealed no significant difference in the mean ΔCp values for patients (*p* = 0.9893), as compared to controls (*p* = 0.7335).

### Analysis of fasting blood sugar, plasma lipid levels, and BMI

**Fig 3:**
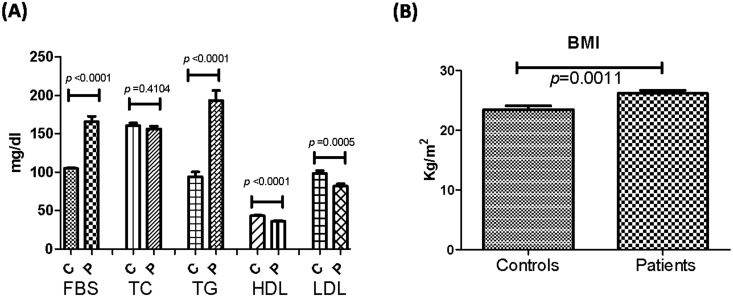
Analysis of fasting blood sugar, plasma lipid levels and BMI in TIID patients and controls. **(A)** Correlation of FBS, total cholesterol, triglycerides, HDL, and LDL levels between controls and TIID patients (p<0.0001, p = 0.4104, p<0.0001, p<0.0001, p = 0.0005). **(B)** Correlation of BMI between controls and TIID patients (p = 0.0011).

Fasting blood sugar, triglycerides (TG), low-density lipoprotein (LDL), and body mass index (BMI) were significantly higher in diabeticswhereas, high-density lipoprotein (HDL) was significantly lower (*p*<0.0001, *p*<0.0001, *p* = 0.0005, *p* = 0.0011 and, *p*<0.0001 respectively). However, there was no statistical difference in total cholesterol (TC) levels between controls and TIID patients (p = 0.4104) (Fig 3A and 3B).

### Correlation of *NPY* +1128 T/C, *NPY* -399 T/C, and *IL1B* -511 C/T polymorphisms with plasma lipid levels and BMI

**Fig 4:**
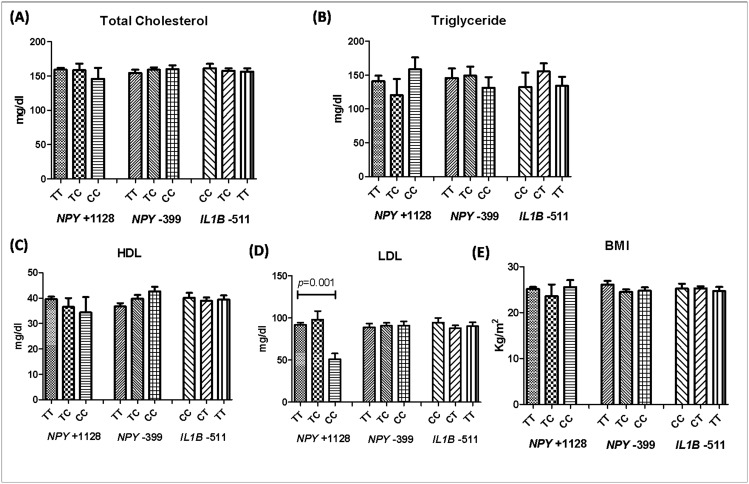
Correlation of *NPY* +1128 T/C, *NPY* -399 T/C, and *IL1B* -511 C/T polymorphisms with plasma lipid concentration and BMI. **(A)** Genotype-phenotype correlation of *NPY* +1128 T/C, *NPY* -399 T/C, and *IL1B* -511 C/T polymorphisms with total cholesterol (p = 0.6798, p = 0.6704, p = 0.8418 respectively). **(B)** Genotype-phenotype correlation of *NPY* +1128 T/C, *NPY* -399 T/C, and *IL1B* -511 C/T polymorphisms with triglycerides (p = 0.8648, p = 0.7037, p = 0.4278 respectively). **(C)** Genotype-phenotype correlation of *NPY* +1128 T/C, *NPY* -399 T/C, and *IL1B* -511 C/T polymorphisms with HDL (p = 0.5064, p = 0.05, p = 0.8936 respectively). **(D)** Genotype-phenotype correlation of *NPY* +1128 T/C, *NPY* -399 T/C, and *IL1B* -511 C/T polymorphisms with LDL (p = 0.01, p = 0.9289, p = 0.6244 respectively). **(E)** Genotype-phenotype correlation of *NPY* +1128 T/C, *NPY* -399 T/C, and *IL1B* -511 C/T polymorphisms with BMI (p = 0.7783, p = 0.2092, p = 0.8016 respectively).

Correlation analysis for *NPY* +1128 T/C SNP revealed increased associated with plasma LDL (*p* = 0.01) levels. However, it was not associated with TC, TG, HDL and BMI (*p* = 0.6798, *p* = 0.8645, *p* = 0.5064, *p* = 0.7783 respectively). Further, *NPY* -399 T/C and *IL1B* -511 did not show any association with plasma lipid (TC, TG, HDL, LDL) or BMI (*p* = 0.6704, *p* = 0.7037, *p* = 0.0560, *p* = 0.9289, *p* = 0.2092; *p* = 0.8418, *p* = 0.4278, *p* = 0.8936, *p* = 0.6244, *p* = 0.8016 respectively) as shown in Fig 4A–4E.

## Discussion

**Fig 5:**
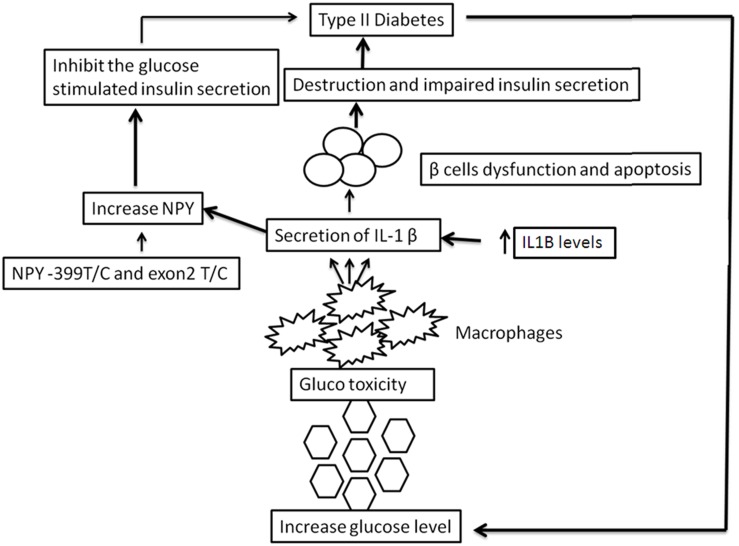
Role of *NPY* -399 T/C, exon 2 T/C and *IL1B* -511 C/T polymorphisms and IL1B in Type-II diabetes. *NPY* -399 T/C, exon 2 T/C SNPs lead to elevated levels of NPY which results in inhibition of glucose-stimulated insulin secretion. Increase in IL1B levels is involved in apoptosis of β cells and impaired insulin secretion and further increase the levels of NPY. Increased blood glucose level causes glucotoxicity which further stimulates macrophages to secrete proinflammatory cytokine IL1B leading to the destruction of β-cells and thereby causing TIID. In Type-II diabetes, chronic hyperglycemia further worsens the condition.

Type-II diabetes results due to lack of functional pancreatic β cell mass following a period of insulin resistance and hyperglycemia. In addition, hyperglycemia has adverse effects on β cells, as the chronic elevation of blood glucose level has been shown to impair β cell function (glucotoxicity) [17]. Increase in IL1B level contributes to apoptosis of β cells and impaired insulin secretion, which in turn leads to increased levels of NPY [17, 12]. The *NPY* -399 T/C, exon 2 +1128 T/C SNPs also contributes to elevated levels of NPY which can result in compromised glucose-stimulated insulin secretion and TIID manifestation [18]. Furthermore, increased glucotoxicity stimulates macrophages to secrete pro-inflammatory cytokines such as IL1B that aggravate β cell destruction (Fig 5). The interaction of NPY and IL1B towards increased pancreatic β cell dysfunction and inhibition of insulin secretion establishes their crucial role in TIID manifestation.

In the present study, we have investigated two polymorphisms of *NPY* which were earlier found to be associated with elevated levels of NPY [19]. Leu7Pro (exon 2 +1128 T/C) polymorphism of *NPY* gene is located in the signal peptide part which influences the processing of preproNPY (prohormone), storage or kinetics of NPY release [20]. Ilhan et al. [21] have shown that diabetic individuals have higher levels of NPY. In addition, this polymorphism was first found to be related with higher lipid levels particularly in obese individuals [8, 22, 23]. Further, the SNP has also been shown to be associated with greater risk for diabetic retinopathy [9,24], diabetic nephropathy [25], and myocardial infarction [26]. Evidently, +1128 T/C polymorphism was shown to have an impact on metabolic, hormonal, and autonomic functions in young healthy subjects [27]. Recently, we have reported the association of *NPY* and *IL1B* polymorphisms with vitiligo susceptibility in Gujarat population [16]. The *NPY* -399 T/C SNP has been shown to exhibit differences in DNA structure and thereby elevate the expression levels of *NPY* [4]. In particular, we found the presence of *NPY* +1128 CC and -399 CC genotypes to be prevalent among TIID patients (Table 1).

Interestingly, the NPY system with a set of molecules also plays an important role in the induction of a number of immune responses by acting on various immune cells [16,28]. In particular, *NPY* +1128 ‘C’ allele has been found to stimulate the production of inflammatory cytokine, IL1B [29].

IL1B, a pro-inflammatory pleiotropic cytokine, is a member of an IL-1 family that has the ability to stimulate the expression of genes responsible for inflammation and immune response. IL1B plays a key role in the pathogenesis of inflammatory and autoimmune diseases [30]. At least three SNPs in the *IL1B* gene have been reported, all representing a C/T base transition at -511 and -31 in the promoter region, and at +3953 in exon 5 [16, 31, 32,]. Camargo et al. [30] have suggested *IL1B* polymorphism to have an effect on risk to acquire Systemic Lupus Erythematosus in the Colombian population. Increased levels of IL1B inhibit β cells within the pancreatic islets leading to destruction and loss of function of these cells [33]. Also, IL1B is reported to stimulate the synthesis and release of NPY which also contribute to induction of type-II diabetes in susceptible subjects [12, 34]. Similarly, there are previous reports showing -511C/T polymorphism to be associated with Alzheimer’s disease [35, 36]. *IL1B* -511 C/T polymorphism was found to be associated with temporal lobe epilepsy (TLE) in hippocampal sclerosis [37, 38], chronic gastritis and gastric ulcer [39], polycystic ovarian syndrome [40], Crohn’s disease [41] and Vitiligo [16].

Achyut et al. [42] have shown a strong association of *IL1B* -511C/T polymorphism with TIID in North Indian populace. However, the present study did not show any association of *IL1B* -511C/T polymorphism with TIID in Gujarat population which might be due to ethnic variation in Indian population (Table 1). One more study in Malaysian population found no association of this promoter polymorphism with TIID supporting the ethnic differences in susceptibility to TIID [43]. On the other hand, *IL1B* transcript levels were upregulated by four-fold in TIID patients as compared to controls (Fig 2A & 2B). O'Neill et al. [44] found that low-grade systemic inflammation exists early in the development of type 2 diabetes and the levels of IL1B and IL6 are augmented in TIID subjects.

Moreover, the haplotype analysis reveals CC and CT haplotype to be more frequently observed in TIID patients suggesting a profound effect of NPY +1128 ‘C’ and NPY -399 ‘C’ alleles (Table 2).

Previously, it has been reported that Leu7Pro substitution in the *NPY* gene has been associated with elevated levels of LDL cholesterol in cardiovascular diseases [45], carotid atherosclerosis [23]. However, Leu7Pro polymorphism was not related to serum LDL-C, HDL-C, and triglyceride concentrations in coronary heart disease sufferers [46]. Schwab et al. [47] revealed that Leu7Pro genotype does not affect BMI and lipid concentrations. Interestingly, our results are in accordance to these where *NPY* +1128 T/C SNP is associated with increased LDL. Also, none of the SNPs studied were found to be associated with BMI, TC, TG, HDL and LDL levels (Fig 4A–4E).

## Conclusion

Our findings suggest that both structural (+1128T/C) and promoter polymorphisms (-399 T/C) of *NPY* are strongly associated with TIID susceptibility in Gujarat population which at least in part, may result in higher levels of NPY thereby suggesting its crucial role in TIID susceptibility. Interestingly, the *NPY* +1128 T/C SNP was found to be associated with increased LDL levels in TIID patients suggesting an important link between these molecules for TIID. Though, *IL1B* promoter (-511 C/T) polymorphism was not found to be associated with TIID, the elevated levels of *IL1B* transcripts observed in patients could confer risk towards TIID. Overall, the study proposes the possible involvement of *NPY* and *IL1B* polymorphisms for genetic susceptibility to TIID in Gujarat population.

## Supporting Information

S1 Table Baseline characteristics of diabetic and non-diabetic individuals from Gujarat population. (DOC)

S2 Table Primers and restriction enzymes used for NPY and IL1B SNPs genotyping and primers for IL1B and GAPDH for mRNA expression studies. (DOC)
