# Supplementary material for: Association of Neuropeptide-Y (NPY) and Interleukin-1beta (IL1B), Genotype-Phenotype Correlation and Plasma Lipids with Type-II Diabetes
Source: PLoS One. 2016 Oct 17;11(10):e0164437. doi: 10.1371/journal.pone.0164437 (PMC5066977; doi:10.1371/journal.pone.0164437)
Supplement: S1 Table — (DOC) [file pone.0164437.s001.doc]

**Table S1. Baseline characteristics of diabetic and non-diabetic individuals from Gujarat population.**

|  | **Non-diabetics**  **(Mean±SD)** |  | **Diabetics**  **(Mean±SD)** | **P value** |
| --- | --- | --- | --- | --- |
| Age  Sex: Male  Female  Fasting blood sugar (mg/dl)  BMI (Kg/m^2^)  Total Cholesterol  Triglycerides  HDL  LDL  Onset age  Duration of disease  Family history | (n =1085) |  | (n =558) |  |
|  | 37.72 ± 17.19 yr  553 (50.96%)  532 (49.04%)  105 ± 10.52  23.74 ± 6.04  160.5 ± 35  94.01 ± 62.33  43.26 ± 11  98.45 ± 35.07  NA  NA  NA |  | 57.29 ± 9.41 yr  293 (52.50%)  265 (47.50%)  177.1 ± 70.97  26.18 ± 5.38  156.4 ± 36.13  193.4 ± 133.3  35.88 ± 12.52  81.85 ± 31.78  50.65 ± 10.10 yr  6.9 ± 6.46 yr  64 (14%) | -  -  -  <0.0001  0.0011  0.4104  <0.0001  <0.0001  0.0005  -  -  - |
