# Supplementary material for: Association of Neuropeptide-Y (NPY) and Interleukin-1beta (IL1B), Genotype-Phenotype Correlation and Plasma Lipids with Type-II Diabetes
Source: PLoS One. 2016 Oct 17;11(10):e0164437. doi: 10.1371/journal.pone.0164437 (PMC5066977; doi:10.1371/journal.pone.0164437)
Supplement: S2 Table — (DOC) [file pone.0164437.s002.doc]

**Table S2.** Primers and restriction enzymes used for *NPY and IL1B* SNPs genotyping and primers for *IL1B* and *GAPDH* for mRNA expression studies.

| **Gene/SNP Primer** | **Sequence** | **Annealing**  **Temperature** | **Amplicon**  **Size (bp) Restriction** | **RestrictionEnzyme** | **Digested products (bp)** |
| --- | --- | --- | --- | --- | --- |
| **(rs16139)**  *NPY* +1128 T/C F  *NPY* +1128 T/C R | 5’- ATTGGGGGTCGCGTGTGGTAG -3’  5’- GTCCTGCCCTGGGATAGAGCG -3’ | 60°C | 402 bp | *BseN*I | 282 bp + 196 bp |
| **(rs16147)**  *NPY* -399 T/C F  *NPY* -399 T/C R | 5’- TTCCTACTCCGGCACCCAGTGAG -3’  5’- GGGCTTTTATGGAGCTTCCTCGC -3’ | 63°C | 417 bp | *Alu*I | 379 bp + 23 bp |
| **(rs16944)**  *IL1B* -511 C/T F  *IL1B* -511 C/T R | 5’- GTTTAGGAATCTTCCCACTT-3’  5’- TGGCATTGATCTGGTTCATC-3’ | 63°C | 305 bp | *Bsu36*I | 192 bp + 113 bp |
| *IL1B* Exp. F  *IL1B* Exp. R | 5’- AGATGAAGTGCTCCTTCCAGG-3’  5’ -TGGTCGGAGATTCGTAGCTG - 3’ | 65°C | 153 bp | - | 153 bp |
| GAPDH Exp. F  GAPDH Exp. R | 5’-ATCCCATCACCATCTTCCAGGA-3  5’-CAAATGAGCCCCAGCCTTCT-3’ | 69°C | 122 bp | - | 122 bp |
